# Authority Without Infrastructure: A Researcher’s Experience With Specimen Access Barriers in Sudden Unexpected Infant Death Research

**DOI:** 10.7759/cureus.117400

**Published:** 2026-10-03

**Authors:** Gary S Goldman

**Affiliations:** 1 Research, Goldman Forensic Research LLC, Bogue Chitto, USA

**Keywords:** biobanking, forensic pathology, forensic research infrastructure, medical examiner systems, medicolegal death investigation, postmortem research governance, postmortem specimen preservation, prospective specimen collection, sudden infant death syndrome (sids), sudden unexpected infant death (suid)

## Abstract

An infant death that remains unexplained after a comprehensive medicolegal investigation does not establish the absence of an underlying biological mechanism. Investigation of a specific research hypothesis may nevertheless be limited when suitable specimens or preservation records are unavailable. This editorial describes non-systematic exploratory outreach undertaken during development of a proposed National Institutes of Health (NIH) R21 study and considers relevant legal provisions in Florida and California.

The outreach identified concerns involving staffing, workflow, authorization, liability, laboratory access, and specimen storage but secured no conditional collection-site commitment. These project-specific observations are hypothesis-generating; they do not establish the frequency of specimen unavailability or a national infrastructure deficiency. A limited, voluntary pilot, potentially using existing research networks, repositories, or academic partnerships, could evaluate prospective specimen collection during routine sudden unexpected infant death (SUID) investigations. It would assess specimen adequacy, workload, cost, and authorization procedures while preserving medicolegal authority and avoiding delays to examinations or body release. Transfer, testing, and secondary use would require appropriate governance. Projects would undergo independent scientific and feasibility review, without priority for the author’s Metabolic Vulnerability Index (MVI) framework. Successful collection would support operational feasibility under the tested conditions, not establish the clinical validity or causal relevance of MVI or other proposed biological mechanisms.

## Editorial

This editorial examines the boundary between forensic investigation and research and considers options for evaluating a prospective sudden unexpected infant death (SUID) specimen-collection pathway.

The unresolved scientific problem

SUID encompasses sudden, unexpected deaths before one year of age, whether subsequently explained or unexplained. Sudden infant death syndrome (SIDS) is a classification applied when death remains unexplained after a comprehensive investigation, including scene investigation, autopsy, histology, toxicology, and clinical-history review [1]. This necessary medicolegal classification marks the limit of the investigation, not the limit of biology. It neither establishes biological homogeneity nor proves that no underlying mechanism exists.

The published Metabolic Vulnerability Index (MVI) framework synthesizes candidate biological vulnerabilities in unexplained infant death and describes a specimen-dependent approach for investigating them [2]. The framework is hypothesis-generating and does not establish that any proposed abnormality contributed to death. Some candidate mechanisms cannot be evaluated retrospectively when required aliquots, fluids, or oriented tissue blocks are not collected at autopsy or promptly preserved for the intended analyses, or when essential handling metadata are not recorded [2].

Table 1 summarizes representative evidence supporting the five proposed MVI domains and their application limits; it is not a systematic review. Application of each domain requires appropriate specimen handling, laboratory quality controls, and interpretive reference limits. Evidence supporting individual measurements or biological observations does not validate the proposed MVI scoring rules or combined profile, which require prospective evaluation and external replication.

**Table 1:** Representative evidence and remaining validation needs for the five proposed MVI domains Abbreviations: CRP, C-reactive protein; CYP, cytochrome P450; IL-6, interleukin-6; MVI, Metabolic Vulnerability Index; SIDS, sudden infant death syndrome

Domain	Biological rationale and measurement evidence	Replication and remaining validation needs
1. Hepatic CYP abundance	Developmental studies document age-related CYP abundance and activity; proteomic methods quantify abundance [3].	Developmental evidence does not validate MVI thresholds. Infant postmortem reference limits and assay reproducibility require evaluation; abundance is not equivalent to activity.
2. Inflammatory markers	IL-6 and CRP reflect inflammatory activity. Adult forensic research supports their measurement under specified postmortem conditions [4].	Infant applicability and the combined domain score remain unvalidated. Terminal events, sampling interval, and specimen quality may affect interpretation.
3. Oxidative injury	Published methods quantify markers such as F₂-isoprostanes, reflecting lipid peroxidation [5].	Analytical-method validation does not validate the domain score. Infant postmortem limits and processing artifacts require assessment; these markers do not directly measure mitochondrial function.
4. Medullary findings	Serotonergic pathways participate in autonomic and respiratory regulation; research immunohistochemistry identifies selected neuronal abnormalities [6].	Selected findings were corroborated in a separate Australian SIDS cohort [6]. The proposed MVI scoring procedure, reader agreement, and individual-case specificity remain unvalidated.
5. Xenobiotic exposure	Forensic toxicology measures selected substances using analyte- and matrix-specific validated methods [7].	Established methods do not validate the MVI exposure score. Infant and postmortem interpretation require qualified toxicologic review; detection alone does not establish causation.

Existing forensic authority

Florida law recognizes standardized SUID investigation and expressly contemplates tissue sampling. Florida Statute §383.3362 places sudden, unexpected deaths of infants younger than one year under medical examiner jurisdiction, requires an autopsy within 24 hours or as soon thereafter as feasible, and authorizes a statewide protocol that may specify scene investigation, data, tissue sampling, and other requirements [8].

Florida Statute §406.11 authorizes a district medical examiner to perform or arrange autopsies and laboratory examinations deemed necessary and in the public interest to determine identity or cause or manner of death, or to obtain evidence for forensic examination. For retention or furnishing of body parts for research outside those purposes or the determination of disease, the statute requires next-of-kin notification and approval unless another specified statutory authorization applies [9].

California provides an instructive comparison. Government Code §27491.41 declares a public interest in SIDS research and authorizes a statewide protocol to specify tissue sampling and repository submission. Research access requires a valid scientific interest, state human-subjects approval, and confidentiality protections. The statute also permits nondisfiguring research sampling without responsible-adult consent and provides liability protection for compliant conduct [10]. It facilitates research authority; it does not compel a county office to join an external study.

The cited statutes establish forms of legal authority for investigation and tissue sampling [8-10]. Participation in a prospective research protocol would also require staffing, funding, approvals, specimen-handling procedures, laboratory access, and governance.

Operational barriers exposed by the R21 feasibility effort

The proposed R21 project was designed to test operational feasibility before a larger, adequately powered investigation.

The contemplated R21 would determine whether a predefined specimen set could be collected, preserved, documented, and qualified prospectively during routine autopsies in SUID investigations. It would require neither a separate research autopsy nor specialized MVI testing by the medical examiner’s office. Instead, established collection, fixation, histologic, toxicologic, and analytical techniques would be assembled under prespecified study procedures. Some aliquots, preservation controls, and assays would nevertheless be additional to local practice; whether they could be incorporated without materially disrupting the examination was a central feasibility question.

A functional web-based MVI application captures case-level laboratory results and applies prespecified rules to assess the five domains. Each case remains associated with one medical examiner’s office, while results from designated laboratories map to that case under predefined data and quality-control procedures.

The proposed R21 pilot would enroll 30 cases meeting initial eligibility and specimen-acceptance requirements to test whether protocol-required specimens can be obtained during routine autopsies, appropriately preserved, and reliably analyzed to produce scorable five-domain profiles. Thirty cases constitute a pragmatic initial series for evaluating this workflow and identifying recurring collection, analytical, or interpretive barriers; the sample is not intended to validate the MVI or test biological associations. The investigator-selected ≥75% completion benchmark requires successful completion in a substantial majority of enrolled cases while allowing approximately one-quarter of profiles to remain incomplete during initial implementation. At n = 30, this requires at least 23 complete, quality-control-qualified, scorable profiles (76.7%). If 23 profiles were complete, the two-sided 95% Wilson confidence interval would be approximately 59.1%-88.2%. Progression would depend on the observed complete-profile yield together with acceptable collection burden, cost, and analytical performance. Reasons for failure at each stage would inform protocol refinement and decisions about progression. Attempted collections and specimen-qualification failures would be reported separately, so that the complete-profile yield among enrolled cases does not obscure barriers to obtaining suitable specimens. All enrolled cases would remain in the feasibility denominator, including cases with incomplete testing or a cause of death subsequently established by the responsible medical examiner. No particular abnormality, biological pattern, or exposure relationship would be required for success. The pilot may identify biological vulnerabilities and physiological constraints worth investigating as possible contributors to death. Still, it would not establish their prevalence or causal role or independently determine diagnosis, cause, or manner of death. If feasibility is demonstrated, a larger, adequately powered study could test whether prespecified clinical and exposure-context variables are associated with individual vulnerabilities or recurring combinations of them.

Potential collaborators were sought initially in Mississippi and nearby Louisiana. Outreach then expanded to offices serving large population centers in other states, with particular attention to California and Florida because of their statutory provisions relevant to SUID investigation and research. This was a purposive search for potential collection sites, not a sampling strategy designed to produce a representative assessment of U.S. forensic offices. Exploratory outreach extended to medical examiner, coroner, and forensic pathology offices in Arizona, California, Florida, Illinois, Louisiana, Maryland, Michigan, Mississippi, Missouri, New Mexico, Ohio, and South Carolina, including offices serving major metropolitan areas and multicounty jurisdictions. The inquiries sought to assess prospective specimen collection, eligible case volume, and practical requirements for participation. Some recipients requested further discussion of study feasibility, but these preliminary exchanges did not constitute commitments to participate.

The collection protocol would distinguish specimens and assays required for five-domain scoring from separately reported exploratory measurements. Any additional exploratory collection would be specified and assessed separately for feasibility and workload.

The governmental contacts described below served an administrative referral function and did not constitute institutional support or endorsement of the proposed project. The inquiry proceeded through a formal referral pathway from the U.S. Department of Health and Human Services (HHS) to the Florida State Surgeon General, the Florida Department of Health, and staff supporting the Florida Medical Examiners Commission. Commission staff provided district contacts and advised that participation must be pursued voluntarily with individual offices because the commission could distribute information but could not compel participation. The referrals and preliminary discussions described here did not constitute endorsement of the MVI framework, the contemplated R21 project, or the recommendations presented in this editorial.

From July through September 2026, the author sent exploratory inquiries to medical examiner offices, coroner offices, forensic autopsy services, and related programs across the listed states. Initial outreach letters contained substantially the same requests, although subsequent exchanges varied according to each recipient’s response. In Florida, inquiries were directed to the chief medical examiners serving all 25 medical examiner districts. Responses representing nine districts (36%) identified operational, administrative, or eligible-case-volume barriers to participation. However, a complete tally of distinct offices contacted, responding, or declining participation in other states was not available; therefore, combined numerical response counts and rates from all states are not reported. Several respondents requested additional information about the proposed study, including the R21 project's Specific Aims and Research Strategy.

Original correspondence was retained separately, and a working feasibility and implementation register distinguished directly communicated concerns and administrative guidance from investigator-derived planning implications and unresolved site-specific questions. Nonresponse was not classified as refusal, and concerns were attributed to respondents only when specifically communicated. The register supported the Florida district-level summary but did not provide a comprehensive multistate contact-and-response dataset or establish barrier frequencies.

Across the jurisdictions included in this exploratory outreach, no conditional operational commitment for a collection site was obtained. This outcome documents recruitment difficulty for the proposed project under its requirements and application timetable. The observations are hypothesis-generating: they suggest that a coordinated prospective collection pathway warrants evaluation, but do not establish a generalized deficiency in the U.S. forensic system or demonstrate that a national pathway is necessary. The legal comparison is limited to Florida and California and does not establish that identical barriers apply elsewhere.

Where applicable law or institutional policy requires next-of-kin authorization, consent coordination may create an additional time-sensitive barrier. Families may welcome an opportunity to contribute to research, yet arranging a sensitive discussion and obtaining authorization soon after an unexpected infant death can require substantial staff time and may not be feasible within the specimen-preservation window. Waiting for authorization may delay research collection or prevent the acquisition of suitable specimens, while delaying body release may add distress for families. Consent logistics may therefore remain a limiting barrier even when collection itself is brief, particularly when authorization must be obtained promptly without pressuring bereaved families or disrupting timely body release.

Any prospective pathway must distinguish medicolegal specimen collection from research-specific collection and secondary research use; authority for the investigation does not itself establish research authorization. Sites should obtain a documented institutional determination of institutional review board (IRB) applicability and resolve jurisdiction-specific consent and privacy requirements. Written agreements should govern custody, access, transfer, retention, and secondary use while preserving evidentiary integrity and avoiding delays to the investigation or body release. A predefined return-of-findings policy should require appropriate validation and qualified review; unvalidated MVI scores should not be reported as diagnoses or causes of death.

The outreach assessed practical feasibility; it was not a systematic survey, human-subjects study, or clinical investigation. It cannot establish the frequency of particular barriers, explain nonresponses, or demonstrate uniformity among jurisdictions.

Statewide protocol change was not pursued as a practical route within the proposed application timetable. Testing the project’s operational feasibility would require willing sites and agreed arrangements for staffing, compensation, materials, authorization, and coordination, without delaying medicolegal duties.

Defining the boundary between forensic investigation and research

Mechanistic assays are scientifically related to unexplained infant death when they evaluate disease, toxic exposure, or biological vulnerabilities not resolved by conventional examination. The legal and operational question is whether particular collection and testing activities are sufficiently connected to the individual death investigation to fall within existing forensic authority.

That determination depends on the medical examiner’s purpose, applicable law, and institutional policy. A research-derived finding may prompt further investigation, but its use in medicolegal conclusions would require documented specimen identity and chain of custody, an analytically valid assay suitable for the specimen and intended interpretation, appropriate confirmatory testing, and compliance with applicable evidentiary requirements. The responsible medical examiner would assess the confirmed finding alongside the complete case evidence; an exploratory MVI score alone would not establish a contributing condition or cause or manner of death.

Returning validated findings may provide forensic value in an individual case, but it does not automatically convert a research assay into routine medicolegal practice. The responsible medical examiner and applicable legal and institutional authorities must define that boundary. The R21 feasibility project is not designed to make that determination; it evaluates whether the proposed specimen and analytical workflow can be implemented once the necessary boundaries and approvals have been established.

Implementation challenges encountered during feasibility outreach

During the author’s feasibility outreach, responsibility and funding for the proposed study involved multiple offices, agencies, and institutions. This effort did not identify a pathway available to the project that coordinated collection kits, training, authorization, storage, transport, testing, specimen governance, and return-of-findings arrangements; it was not a systematic assessment of infrastructure available nationally. A central practical challenge was timing: the required specimens must be collected during autopsy and promptly preserved. Later approvals cannot recover specimens that were never collected or restore the quality of specimens that were inadequately preserved.

Options for evaluating a prospective collection pathway

Potential approaches include strengthening existing SUID research networks, partnering with state repositories or academic institutions, and adapting established forensic tissue-collection programs. The author’s outreach did not systematically assess these alternatives or establish that a new pilot would be preferable. A limited, voluntary pilot could operate through existing programs where feasible, testing whether they can support the proposed specimen requirements, authorization procedures, and collection timetable. The choice should depend on available capabilities, participating-site interest, cost, and workload.

The proposed R21 project specifies the following feasibility outcomes: added collection time, specimen-acquisition and qualification yields, authorization timing, preservation and transfer performance, laboratory evaluability, site burden, and cost. These could serve as the pilot’s principal measures. A prespecified process should also govern communication of validated, potentially relevant individual findings to the responsible medical examiner when scientifically and legally appropriate.

Residual specimens could support future research only within the scope of applicable authorization and institutional approvals. A designated institutional custodian would oversee access, with scientific and ethics review as applicable and written agreements limiting permitted uses and onward transfer. Secondary-use authorization would specify the scope of future research, a defined retention period, and procedures for destruction or other authorized disposition. Family preferences and any limits on withdrawal after distribution or analysis would be documented and explained; uses outside the authorized scope would require additional authorization or another applicable legal basis. Within these safeguards, linked clinical and exposure data could support studies of reproducible associations without treating temporal proximity alone as evidence of causation.

Any proposed collection pathway should be evaluated against the needs of multiple qualified SUID research projects, with demonstration projects selected through independent scientific and feasibility review. The MVI requirements discussed here illustrate one project’s needs; they should not define a general collection standard or confer priority for funding or specimen access.

The medical examiner would retain authority over the investigation, autopsy, specimen custody, body release, classification, and certification. Research personnel would not direct the examination or require the office to perform specialized research testing. The pilot would provide the evidence needed to decide whether the approach should stop, continue narrowly, or expand into a broader shared SUID research resource.

Evaluating a prospective collection pathway

Mechanistic research in unexplained infant death depends on appropriate specimens collected and preserved at autopsy. Once required material is discarded or becomes unsuitable for analysis, some research questions can no longer be investigated in that case. Florida and California have statutory provisions concerning SUID investigation and tissue sampling [8-10]. The author’s exploratory outreach did not secure a conditional commitment from any office to serve as a collection site for the proposed project.

This experience documented recruitment difficulties before enrollment; it did not establish how often suitable specimens are unavailable or demonstrate a national infrastructure deficiency. A limited, voluntary pilot could test whether a coordinated collection pathway can obtain research-quality specimens at an acceptable cost and workload, with appropriate authorization and without disrupting medicolegal responsibilities. Such a pilot would evaluate one possible response to the barriers encountered rather than presume that a new national pathway is necessary.
